# 4-(3-Carb­oxy­phen­yl)pyridinium nitrate

**DOI:** 10.1107/S1600536812013918

**Published:** 2012-04-06

**Authors:** Long Tang, Ya-Pan Wu, Feng Fu, Zhu-Lian Zhang, Hai-Kang Guo

**Affiliations:** a Department of Chemistry and Chemical Engineering, Shaanxi Key Laboratory of Chemical Reaction Engineering, Yan’an University, Yan’an, Shaanxi 716000, People’s Republic of China

## Abstract

In the title salt, C_12_H_10_NO_2_^+^·NO_3_^−^, the dihedral angle between the pyridine ring and the benzene ring of the 4-(3-carb­oxy­phen­yl)pyridinium cation is 30.14 (2)°. Inversion-related pairs of cations are linked into dimers by pairs of O—H⋯O hydrogen bonds. Pairs of dimers are linked by N—H⋯O and C—H⋯O hydrogen bonds involving nitrate anions as acceptors, generating supra­molecular chains along the diagonal of the *bc* plane.

## Related literature

For structures of similar compounds, see: Jin *et al.* (2003 ▶); Bei *et al.* (2004 ▶); Rademeyer (2005 ▶); Wang (2006 ▶); Yu *et al.* (2006 ▶).
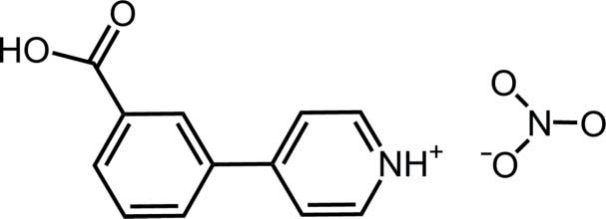


## Experimental

### 

#### Crystal data

C_12_H_10_NO_2_^+^·NO_3_^−^
*M* *_r_* = 262.22
Triclinic, 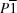

*a* = 5.2545 (11) Å
*b* = 7.0617 (14) Å
*c* = 16.469 (3) Å
α = 97.39 (3)°
β = 92.96 (5)°
γ = 106.05 (3)°
*V* = 580.0 (2) Å^3^
*Z* = 2
Mo *K*α radiation
μ = 0.12 mm^−1^
*T* = 293 K
0.15 × 0.13 × 0.10 mm

#### Data collection

Bruker SMART diffractometer
Absorption correction: multi-scan (*SADABS*; Sheldrick, 1996 ▶) *T*_min_ = 0.982, *T*_max_ = 0.988
4634 measured reflections
2255 independent reflections
1599 reflections with *I* > 2σ(*I*)
*R*_int_ = 0.042

#### Refinement

*R*[*F* ^2^ > 2σ(*F* ^2^)] = 0.050
*wR*(*F* ^2^) = 0.127
*S* = 1.00
2255 reflections
174 parameters
H-atom parameters constrained
Δρ_max_ = 0.20 e Å^−3^
Δρ_min_ = −0.22 e Å^−3^

### 

Data collection: *SMART* (Bruker, 1997 ▶); cell refinement: *SAINT* (Bruker, 1997 ▶); data reduction: *SAINT*; program(s) used to solve structure: *SHELXS97* (Sheldrick, 2008 ▶); program(s) used to refine structure: *SHELXL97* (Sheldrick, 2008 ▶); molecular graphics: *SHELXTL* (Sheldrick, 2008 ▶); software used to prepare material for publication: *SHELXTL*.

## Supplementary Material

Crystal structure: contains datablock(s) I, global. DOI: 10.1107/S1600536812013918/pk2401sup1.cif

Structure factors: contains datablock(s) I. DOI: 10.1107/S1600536812013918/pk2401Isup2.hkl

Supplementary material file. DOI: 10.1107/S1600536812013918/pk2401Isup3.cml

Additional supplementary materials:  crystallographic information; 3D view; checkCIF report

## Figures and Tables

**Table 1:** Hydrogen-bond geometry (Å, °)

*D*—H⋯*A*	*D*—H	H⋯*A*	*D*⋯*A*	*D*—H⋯*A*
C10—H10⋯O4^i^	0.93	2.42	3.225 (3)	145
C11—H11⋯O5^ii^	0.93	2.49	3.122 (3)	125
O1—H1⋯O2^iii^	0.82	1.82	2.624 (2)	167
N2—H2⋯O4^ii^	0.86	1.89	2.748 (3)	174

Symmetry codes: (i) 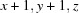; (ii) 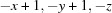; (iii) 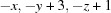.

**Fig. 1.**
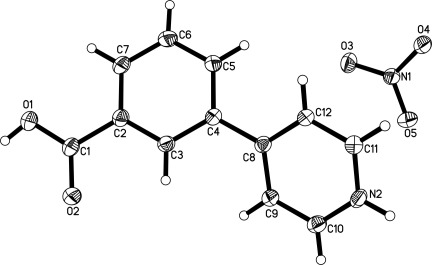
Thermal ellipsoid plot of the title compound (30% probability level), hydrogen atoms are drawn as spheres of arbitrary radius.

**Fig. 2.**
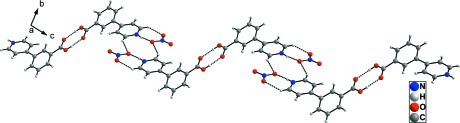
The one-dimensional hydrogen-bonded supramolecular chains in the crystal structure. Dashed lines denote hydrogen bonds.

## supplementary crystallographic information

### Comment

The title compound consists of a 3-(pyridin-4-yl)benzoic acid cation and a nitrate anion (Fig. 1). The nitric acid is deprotonated and the pyridine ring accepts the proton to produce the protonated organic cation, namely 3-(pyridin-4-yl)benzoic acid nitrate. The dihedral angle between pyridine ring and the benzene ring of the 3-(pyridin-4-yl)benzoic acid is 30.14 (2)°. The two components are linked by O—H···O [O1—H1···O2, 2.624 (2) Å] hydrogen bonds to form the cation dimers (Fig. 2), and further though N—H···O and C—H···O [N2—H2···O4, 2.748 (3) Å; C10—H10···O4, 3.225 (3) Å; C11—H11···O5, 3.122 (3)Å] hydrogen bonds. The cation dimers are thus connected by the nitrate anions into extended one-dimensional supramolecular chains (Fig. 2).

### Experimental

The title compound was prepared by a hydrothermal method. An aqueous solution (20 mL) containing 3-(pyridin-4-yl)benzoic acid (0.10 mmol) and samarium nitrate hexahydrate (0.10 mmol) was placed in a Parr Teflon-lined stainless steel vessel (25 mL) under autogenous pressure, and then heated to 433 K for 72 h and subsequently cooled to room temperature at a rate of 5 K an hour. The targeted Sm^3+^ complex was not synthesized. Unintentionally, colorless single crystals of the title compound suitable for X-ray analysis were obtained from the reaction mixture.

### Refinement

All H atoms were positioned geometrically (C-H = 0.93Å, O-H = 0.82 Å and N-H = 0.86 Å) and allowed to ride on their parent atoms, with *U*_iso_(H) values equal to 1.2*U*_eq_(C, N) or 1.5*U*_eq_(O).

### Crystal data

**Table:**

C_12_H_10_NO_2_^+^·NO_3_^−^	*Z* = 2
*M**_r_* = 262.22	*F*(000) = 272
Triclinic, *P*1	*D*_x_ = 1.502 Mg m^−^^3^
Hall symbol: -P 1	Mo *K*α radiation, λ = 0.71073 Å
*a* = 5.2545 (11) Å	Cell parameters from 2373 reflections
*b* = 7.0617 (14) Å	θ = 3.0–27.5°
*c* = 16.469 (3) Å	µ = 0.12 mm^−^^1^
α = 97.39 (3)°	*T* = 293 K
β = 92.96 (5)°	Block, colourless
γ = 106.05 (3)°	0.15 × 0.13 × 0.10 mm
*V* = 580.0 (2) Å^3^	

### Data collection

**Table:**

Bruker SMART diffractometer	2255 independent reflections
Radiation source: fine-focus sealed tube	1599 reflections with *I* > 2σ(*I*)
Graphite monochromator	*R*_int_ = 0.042
φ and ω scans	θ_max_ = 26.0°, θ_min_ = 3.0°
Absorption correction: multi-scan (*SADABS*; Sheldrick, 1996)	*h* = −4→6
*T*_min_ = 0.982, *T*_max_ = 0.988	*k* = −8→8
4634 measured reflections	*l* = −20→17

### Refinement

**Table:**

Refinement on *F*^2^	Secondary atom site location: difference Fourier map
Least-squares matrix: full	Hydrogen site location: inferred from neighbouring sites
*R*[*F*^2^ > 2σ(*F*^2^)] = 0.050	H-atom parameters constrained
*wR*(*F*^2^) = 0.127	*w* = 1/[σ^2^(*F*_o_^2^) + (0.063*P*)^2^] where *P* = (*F*_o_^2^ + 2*F*_c_^2^)/3
*S* = 1.00	(Δ/σ)_max_ < 0.001
2255 reflections	Δρ_max_ = 0.20 e Å^−^^3^
174 parameters	Δρ_min_ = −0.22 e Å^−^^3^
0 restraints	Extinction correction: *SHELXL97* (Sheldrick, 2008), Fc^*^=kFc[1+0.001xFc^2^λ^3^/sin(2θ)]^-1/4^
Primary atom site location: structure-invariant direct methods	Extinction coefficient: 0.039 (7)

### Special details

**Table:**

Geometry. All esds (except the esd in the dihedral angle between two l.s. planes) are estimated using the full covariance matrix. The cell esds are taken into account individually in the estimation of esds in distances, angles and torsion angles; correlations between esds in cell parameters are only used when they are defined by crystal symmetry. An approximate (isotropic) treatment of cell esds is used for estimating esds involving l.s. planes.
Refinement. Refinement of F^2^ against ALL reflections. The weighted R-factor wR and goodness of fit S are based on F^2^, conventional R-factors R are based on F, with F set to zero for negative F^2^. The threshold expression of F^2^ > 2σ(F^2^) is used only for calculating R-factors(gt) etc. and is not relevant to the choice of reflections for refinement. R-factors based on F^2^ are statistically about twice as large as those based on F, and R- factors based on ALL data will be even larger.

### Fractional atomic coordinates and isotropic or equivalent isotropic displacement parameters (Å^2^)

**Table:**

	*x*	*y*	*z*	*U*_iso_*/*U*_eq_	
N2	0.5337 (3)	0.8423 (2)	0.07114 (11)	0.0357 (4)	
H2	0.6254	0.8387	0.0295	0.043*	
O2	0.1639 (3)	1.3909 (2)	0.43108 (10)	0.0541 (5)	
O1	−0.2183 (3)	1.2586 (2)	0.48279 (11)	0.0555 (5)	
H1	−0.1765	1.3685	0.5113	0.083*	
C4	0.0785 (4)	0.8582 (3)	0.27490 (12)	0.0316 (5)	
C8	0.2414 (4)	0.8503 (3)	0.20449 (13)	0.0306 (5)	
C12	0.1417 (4)	0.7172 (3)	0.13261 (13)	0.0352 (5)	
H12	−0.0275	0.6284	0.1292	0.042*	
C10	0.6380 (4)	0.9746 (3)	0.13859 (13)	0.0370 (5)	
H10	0.8073	1.0623	0.1400	0.044*	
C9	0.4953 (4)	0.9804 (3)	0.20526 (13)	0.0341 (5)	
H9	0.5685	1.0726	0.2519	0.041*	
C3	0.1074 (4)	1.0373 (3)	0.32576 (12)	0.0332 (5)	
H3	0.2408	1.1501	0.3185	0.040*	
C1	−0.0351 (4)	1.2460 (3)	0.43713 (14)	0.0394 (5)	
C5	−0.1169 (4)	0.6893 (3)	0.28921 (14)	0.0384 (5)	
H5	−0.1356	0.5674	0.2568	0.046*	
C11	0.2897 (4)	0.7157 (3)	0.06729 (14)	0.0384 (5)	
H11	0.2208	0.6260	0.0196	0.046*	
C7	−0.2581 (4)	0.8815 (3)	0.39976 (14)	0.0410 (5)	
H7	−0.3726	0.8898	0.4404	0.049*	
C2	−0.0603 (4)	1.0497 (3)	0.38714 (13)	0.0349 (5)	
C6	−0.2820 (5)	0.7011 (3)	0.35072 (14)	0.0454 (6)	
H6	−0.4108	0.5872	0.3595	0.054*	
O3	0.2280 (3)	0.3289 (2)	0.18749 (10)	0.0466 (4)	
O5	0.5525 (3)	0.3893 (2)	0.10947 (10)	0.0513 (5)	
N1	0.3185 (4)	0.3064 (2)	0.12032 (11)	0.0355 (4)	
O4	0.1696 (3)	0.1940 (2)	0.05948 (10)	0.0479 (5)	

### Atomic displacement parameters (Å^2^)

**Table:**

	*U*^11^	*U*^22^	*U*^33^	*U*^12^	*U*^13^	*U*^23^
N2	0.0348 (10)	0.0396 (9)	0.0369 (11)	0.0138 (7)	0.0154 (8)	0.0091 (8)
O2	0.0551 (11)	0.0408 (9)	0.0583 (11)	0.0013 (7)	0.0264 (9)	−0.0027 (7)
O1	0.0538 (11)	0.0479 (10)	0.0593 (12)	0.0076 (8)	0.0287 (9)	−0.0062 (8)
C4	0.0293 (11)	0.0351 (11)	0.0310 (12)	0.0097 (8)	0.0049 (9)	0.0057 (8)
C8	0.0289 (11)	0.0306 (10)	0.0345 (12)	0.0104 (8)	0.0059 (9)	0.0075 (8)
C12	0.0317 (11)	0.0327 (10)	0.0390 (13)	0.0057 (8)	0.0102 (10)	0.0017 (9)
C10	0.0311 (12)	0.0372 (11)	0.0440 (14)	0.0093 (9)	0.0066 (10)	0.0102 (10)
C9	0.0304 (11)	0.0353 (10)	0.0354 (12)	0.0077 (8)	0.0055 (9)	0.0038 (9)
C3	0.0312 (11)	0.0341 (10)	0.0318 (12)	0.0051 (8)	0.0053 (9)	0.0047 (9)
C1	0.0409 (13)	0.0431 (12)	0.0346 (12)	0.0113 (10)	0.0125 (10)	0.0052 (9)
C5	0.0415 (13)	0.0315 (11)	0.0394 (13)	0.0054 (9)	0.0124 (10)	0.0032 (9)
C11	0.0383 (13)	0.0349 (11)	0.0398 (13)	0.0077 (9)	0.0090 (10)	0.0015 (9)
C7	0.0411 (13)	0.0458 (12)	0.0350 (13)	0.0081 (10)	0.0160 (10)	0.0068 (10)
C2	0.0353 (12)	0.0375 (11)	0.0316 (12)	0.0097 (9)	0.0064 (10)	0.0041 (9)
C6	0.0453 (14)	0.0393 (12)	0.0457 (14)	−0.0001 (10)	0.0159 (11)	0.0071 (10)
O3	0.0551 (11)	0.0481 (9)	0.0378 (10)	0.0147 (7)	0.0171 (8)	0.0054 (7)
O5	0.0345 (10)	0.0517 (9)	0.0570 (11)	−0.0034 (7)	0.0119 (8)	0.0011 (8)
N1	0.0376 (11)	0.0296 (9)	0.0397 (11)	0.0085 (7)	0.0093 (9)	0.0061 (8)
O4	0.0348 (9)	0.0587 (10)	0.0393 (9)	0.0008 (7)	0.0076 (7)	−0.0064 (7)

### Geometric parameters (Å, º)

**Table:**

N2—C11	1.338 (3)	C9—H9	0.9300
N2—C10	1.339 (3)	C3—C2	1.385 (3)
N2—H2	0.8600	C3—H3	0.9300
O2—C1	1.263 (3)	C1—C2	1.487 (3)
O1—C1	1.267 (3)	C5—C6	1.377 (3)
O1—H1	0.8200	C5—H5	0.9300
C4—C3	1.391 (3)	C11—H11	0.9300
C4—C5	1.398 (3)	C7—C6	1.388 (3)
C4—C8	1.481 (3)	C7—C2	1.393 (3)
C8—C12	1.392 (3)	C7—H7	0.9300
C8—C9	1.393 (3)	C6—H6	0.9300
C12—C11	1.360 (3)	O3—N1	1.235 (2)
C12—H12	0.9300	O5—N1	1.240 (2)
C10—C9	1.364 (3)	N1—O4	1.272 (2)
C10—H10	0.9300		
			
C11—N2—C10	121.3 (2)	O2—C1—O1	123.68 (19)
C11—N2—H2	119.4	O2—C1—C2	118.7 (2)
C10—N2—H2	119.4	O1—C1—C2	117.61 (19)
C1—O1—H1	109.5	C6—C5—C4	120.80 (19)
C3—C4—C5	118.4 (2)	C6—C5—H5	119.6
C3—C4—C8	120.40 (18)	C4—C5—H5	119.6
C5—C4—C8	121.07 (17)	N2—C11—C12	120.49 (19)
C12—C8—C9	116.8 (2)	N2—C11—H11	119.8
C12—C8—C4	121.07 (18)	C12—C11—H11	119.8
C9—C8—C4	122.08 (18)	C6—C7—C2	119.0 (2)
C11—C12—C8	120.60 (19)	C6—C7—H7	120.5
C11—C12—H12	119.7	C2—C7—H7	120.5
C8—C12—H12	119.7	C3—C2—C7	120.33 (18)
N2—C10—C9	119.82 (19)	C3—C2—C1	119.46 (18)
N2—C10—H10	120.1	C7—C2—C1	120.2 (2)
C9—C10—H10	120.1	C5—C6—C7	120.6 (2)
C10—C9—C8	121.00 (19)	C5—C6—H6	119.7
C10—C9—H9	119.5	C7—C6—H6	119.7
C8—C9—H9	119.5	O3—N1—O5	122.40 (18)
C2—C3—C4	120.76 (18)	O3—N1—O4	119.71 (18)
C2—C3—H3	119.6	O5—N1—O4	117.89 (19)
C4—C3—H3	119.6		
			
C3—C4—C8—C12	−147.6 (2)	C8—C4—C5—C6	−174.7 (2)
C5—C4—C8—C12	28.9 (3)	C10—N2—C11—C12	−0.6 (3)
C3—C4—C8—C9	29.6 (3)	C8—C12—C11—N2	0.0 (3)
C5—C4—C8—C9	−154.0 (2)	C4—C3—C2—C7	1.1 (3)
C9—C8—C12—C11	0.5 (3)	C4—C3—C2—C1	−176.26 (19)
C4—C8—C12—C11	177.82 (19)	C6—C7—C2—C3	0.9 (3)
C11—N2—C10—C9	0.6 (3)	C6—C7—C2—C1	178.2 (2)
N2—C10—C9—C8	0.0 (3)	O2—C1—C2—C3	−10.7 (3)
C12—C8—C9—C10	−0.6 (3)	O1—C1—C2—C3	168.3 (2)
C4—C8—C9—C10	−177.83 (18)	O2—C1—C2—C7	172.0 (2)
C5—C4—C3—C2	−2.4 (3)	O1—C1—C2—C7	−9.0 (3)
C8—C4—C3—C2	174.15 (18)	C4—C5—C6—C7	0.1 (4)
C3—C4—C5—C6	1.8 (3)	C2—C7—C6—C5	−1.5 (4)

### Hydrogen-bond geometry (Å, º)

**Table:**

*D*—H···*A*	*D*—H	H···*A*	*D*···*A*	*D*—H···*A*
C10—H10···O4^i^	0.93	2.42	3.225 (3)	145
C11—H11···O5^ii^	0.93	2.49	3.122 (3)	125
O1—H1···O2^iii^	0.82	1.82	2.624 (2)	167
N2—H2···O4^ii^	0.86	1.89	2.748 (3)	174

Symmetry codes: (i) *x*+1, *y*+1, *z*; (ii) −*x*+1, −*y*+1, −*z*; (iii) −*x*, −*y*+3, −*z*+1.
